# Physiological responses of small-sided vs. regular games in youth volleyball players

**DOI:** 10.5114/biolsport.2023.114291

**Published:** 2022-05-10

**Authors:** Jamel Halouani, Cyrine H’mida, Khaled Trabelsi, Cain C.T Clark, Jordan M. Glenn, Hamdi Chtourou

**Affiliations:** 1 Research Laboratory, Education, Motricity, Sport and Health, LR19JS01. Higher Institute of Sport and Physical Education of Sfax, University of Sfax, Sfax, Tunisia; 2 Higher Institute of Sport and Physical Education of Gafsa, University of Gafsa, Tunisia; 3 Higher Institute of Sport and Physical Education of Sfax, University of Sfax, Sfax, Tunisia; 4 Centre for Intelligent Healthcare, Coventry University, Coventry, CV1 5FB, UK; 5 Department of Health, Exercise Science Research Center Human Performance and Recreation, University of Arkansas, Fayetteville, Arkansas, USA; 6 Physical Activity, Sport, and Health, UR18JS01, National Observatory of Sport, Tunis, Tunisia

**Keywords:** Small-sided games, Regular game, Physiological responses, Volleyball, Youth

## Abstract

The aim of the present study was to evaluate the effects of two different formats of small-sided games (SSGs; 2 vs. 2 and 3 vs. 3) on physiological parameters in youth volleyball players. Twelve youth volleyball players (17.2 ± 7.44 years, 1.89 ± 0.6 m, 72.83 ± 8.57 kg) completed three different games formats (regular game (RG): 6 vs. 6, SSG3: 3 vs. 3 and SSG2: 2 vs. 2). associated with two pitch dimensions (i.e., 2 vs. 2 and 3 vs. 3 on 18 × 4.5 m; 6 vs. 6 on 18 × 9 m). Each player performed 4 × 5 min SSG with a recovery period of 1 min between bouts. All players were members of the same youth team and played in a professional league. They had at least 6 years of volleyball training and no current injuries. This study was carried out during the competitive period. Heart rate (HR), blood lactate ([La^−^]), and rating of perceived exertion (RPE) were measured. Compared to RG, physiological (i.e., HR and [La^−^]) and RPE responses were significantly higher during SSG3 and SSG2 (all p < 0.05; ƞ_p_^2^ = 0.77, ƞ_p_^2^ = 0.65, ƞ_p_^2^ = 0.30, respectively). Moreover, HR and RPE were significantly higher in SSG2 compared to SSG3. In contrast, no significant differences were observed in [La^−^] between SSG2 and SSG3. These results suggest that the number of players influences the exercise intensity in small-sided volleyball games in youth players. Therefore, coaches could benefit from incorporating SSGs to manipulate the exercise intensity in youth volleyball players.

## INTRODUCTION

Small-sided games (SSGs) are very popular in team sports such as soccer, rugby [1] and Taekwondo [2], because they increase player’s perception of specific tactical/technical issues, while promoting variations in physiological and physical stimuli [3, 4, 5]. Thus, SSGs allow for the identification a players’ skill level, as well as proposing effectual training interventions in contextualized and situational manners [3].

SSGs offer multiple possibilities of factor variations. Indeed, several studies have analyzed the effect of modifying the number of players [6, 7], pitch size [8, 2], area per player [9], exercise duration [10, 11], coach encouragements [12], rule changes [1, 7, 8, 13], ball contacts [14], and different periods of play [15] on the physiological demands of soccer. Indeed, most studies [6, 7] have shown SSGs result in greater heart rate (HR), lactate concentration ([La^−^]), and rating of perceived (RPE) exertion. Also, studies [8, 2] have found increases in physiological parameters (i.e., HR, [La^−^] and RPE), due to needing to cover an increased pitch area. These studies confirm that, by altering these factors, it is possible to manipulate the overall physiological and perceptual workload placed on players. SSGs are increasingly being used to improve skill and physical fitness of team-sport athletes [16]. Given the importance of these physiological and skill qualities to team-sport performance, coaches are motivated to discover the most effective methods of developing these attributes in their athletes [17]. Although SSGs has been shown to provide a specific training stimulus that generally replicates the overall demands of team-sports, recent evidence suggests that it may not always reach levels needed to replicate the high intensity and the demands of competition [18].

Volleyball is an intermittent court sport, with multiple jumps and lateral movements performed throughout a match [19]. It requires well-developed speed, agility, upper- and lower-body muscular power, and maximal aerobic power (V̇O_2max_) [19]. Moreover, volleyball strategy implies a special consideration on game skills efficiency aspects where players must acquire all specific motions: serve, receive, set, attack, block, and dig [20]. Smith et al. [21] suggested that physiological capacities play an important role in the preparation and selection of elite volleyball players. Furthermore, in volleyball, skill-based conditioning games offer a specific training stimulus to simulate the physiological demands of competition in youth volleyball players [22, 23]. Indeed, the improvement in physiological capacities in volleyball players was reportedly greater with the use of skill-based conditioning games vs. instructional training [22, 24, 25]. Gabbett, [22] suggested that conditioning coaches may use skill-based conditioning games during game-specific phases of training to elicit improvements in muscular power, speed, agility, and maximal aerobic power in order to promote the development of game-specific skills under fatigue. Thus, the use of skill-based conditioning games as training drills allows the simulation of movement patterns of team sports, while maintaining a competitive environment in which athletes must perform under pressure and fatigue [26, 27, 23].

To the best of our knowledge, no study has attempted to evaluate the physiological impact (i.e., HR, [La^−^] and RPE) of SSGs in youth volleyball players. Most SSG studies reported in the literature have been conducted in a limited number of team sports (i.e., soccer, rugby, handball, and basketball) [1, 28, 29].

Given that the task constraints manipulation could affect the physiological responses and, subsequently, the magnitude of performance improvement, SSGs may offer an additional challenge to volleyball players that would not normally be present in non–skill-related conditioning activities. The specificity principle dictates that the demands of a particular sport, or the demands of a task in which an athlete try to improve performance, will directly determine the manner in which the training should be performed. In addition, determining which format of SSGs would promote better physiological responses in volleyball is of practical significance. Therefore, the aim of the present study was to compare the effects of two formats of SSGs (SSG2: 2 vs. 2 and SSG3: 3 vs. 3) with a regular game (RG: 6 vs. 6) on exercise intensity in volleyball youth players. We hypothesized that the reduction of player number would elicit increases in the exercise intensity (higher physiological (HR and [La^−^]) and perceptual (RPE) values). The goal of this study is to greater enable volleyball coaches and/or fitness coaches to determine training intensity when adopting SSG formats. Moreover, the findings could potentially provide valuable information to coaches when designing and promoting the use of SSGs training as part of a conditioning program.

## MATERIALS AND METHODS

### Subjects

Sample size was calculated using the software G*power (version 3.1.9.7; Kiel University, Kiel, Germany) with α = 0.05 and power = 0.80 and based on the effect size (ES = 0.4) reported by Praça et al. [30]. A minimum of 10 participants for each group was required for inclusion in the present study. Due to potential dropout, 12 participants were recruited.

Twelve young volleyball players (age: 17.2 ± 7.44 years; height: 18.9 ± 0.05 m; body mass: 72.83 ± 8.57 kg) voluntarily participated in this study. All players were members of the same youth team and played in a professional league. They had an experience of at least 6 years of volleyball training. Subjects were chosen by the responsible technical committee, so that the team were balanced regarding the positions of the players (setter, outside hitter, opposite hitter, middle blocker, libero and defensive specialist). This precaution was taken to allow the team to maintain a high level of competitiveness and concentration during the games. Substitute players and those with injuries were excluded from the study. All the players and their parents/legal guardians were notified about the research design and its requirements, as well as the potential benefits and risks. Each participant gave written informed consent prior to the start. The study was approved by the local ethics committee, and was conducted in a manner consistent with the institutional ethical requirements for human experimentation in accordance with the Declaration of Helsinki 1964 and its further amendments.

### Experimental procedure

To investigate the effect of two volley-ball SSGs formats (i.e., SSG2: 2 vs. 2 and SSG3: 3 vs. 3) with RG (i.e., 6 vs. 6) on physiological responses: two pitch dimensions and three formats were employed [i.e., 2 vs. 2 and 3 vs. 3 on 18 × 4.5 m (81 m^2^); 6 vs. 6 on 18 × 9 m (162 m^2^)]. The pitch ratio per player (pitch area divided by the number of players: m^2^ × player) [31] was 1:20.2 m^2^, 1:13.5 m^2^ and 1:13.5 m^2^, respectively for 2 vs. 2, 3 vs. 3 and 6 vs. 6. Each player performed 4 × 5 min SSG with a recovery period of 1 min between bouts. The training intervention was conducted during the competition period from 2019–2020. Participants usually trained using the SSGs with varying numbers of players; but they have been further familiarized with the specific SSG formats (i.e., 2 vs. 2; 3 vs. 3 and 6 vs. 6) during three weeks before the experiment. A physician staff recommended and monitored the dietary regime and hydration status of each player before and after every training session.

**FIG. 1:**
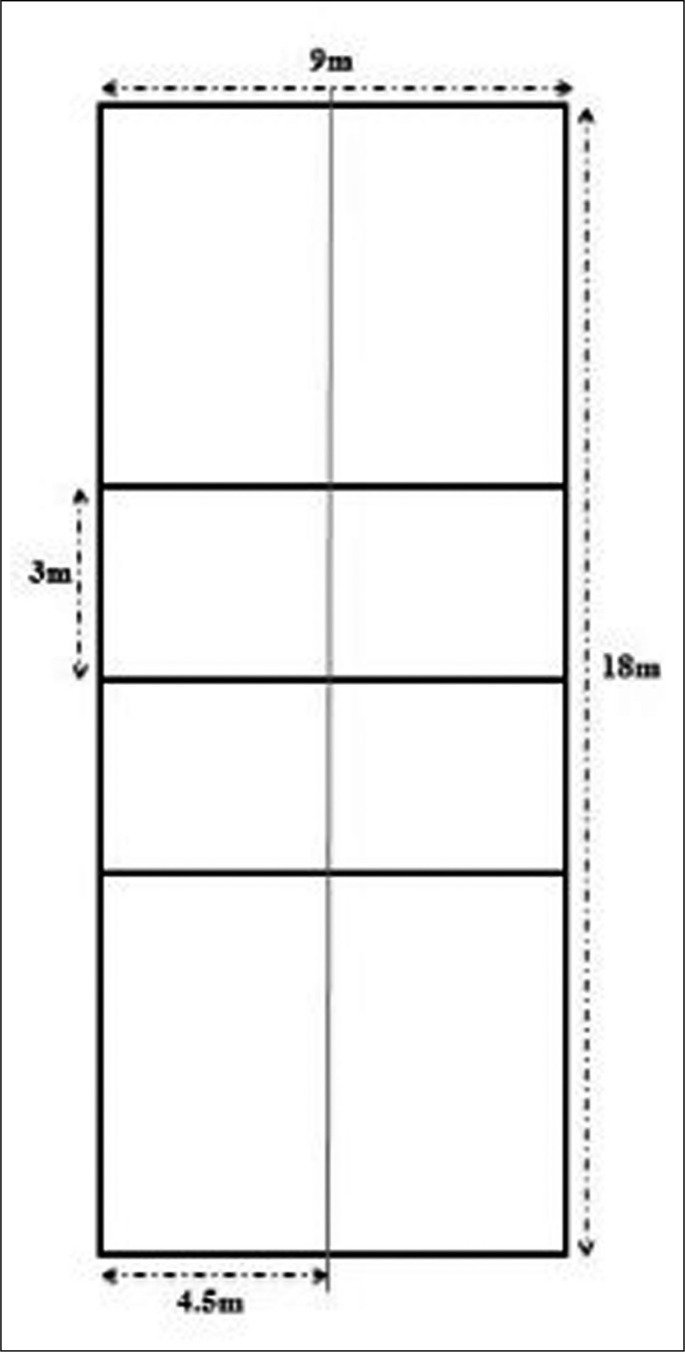
Volleyball court for SSGs.

The SSGs consisted of teams of 2 or 3 outfield players being played on 18 × 4.5 m pitch, whereas the RG consisted of 6 players a-side played on 18 × 9 m pitch. Players were divided regarding their positions (setter, outside hitter, opposite hitter, middle blocker, libero and defensive specialist). Both SSGs had the same duration and lasted for 4 × 5 min with 1 min of passive recovery between games, while the RG consisted of a set of 25 points with two technical timeout of 1 min and 2 voluntary time-out. Moreover, both SSGs and RG involved normal match rules with no other added conditions. No specific tactical instructions were imposed to players within the games. The SSGs were performed under the supervision of their coach and the responsible technical committee to keep up a high work rate. For this reason, a large number of balls were placed near to the net to ensure a continued play (figure 1). Each game was preceded by a 20-min standardized warm-up consisting of low-intensity running, striding, and dynamic stretching with a final 5 min of ball passing. All games were played at the beginning of the training sessions. The order of RG on Monday in the third week, SSG3 on Monday in the fourth week, and SSG2 on Monday in the fifth week of the investigation. As requested by the coach, SSGs were not scheduled the day before a competitive game.

Heart rate (HR) was continuously monitored throughout the SSGs and the RG by HR monitors (Polar Team Sport System, Polar-Electro OY, Kempele, Finland) and recorded at 5-s intervals. Individual mean HR during RG and SSGs were determined to indicate the overall intensity. HR data were therefore expressed as percentage of HR_max_ (%HR_max_). Capillary blood samples were taken from an earlobe at one minute from the end of the last bout of SSG and RG [32]; and immediately analyzed for lactate using a portable amperometric microvolume lactate analyzer (Lactate Pro, Arkray, Japan). RPE scores (10-point scale [33]) were recorded immediately after the end of exercises (SSG2, SSG3 and RG) to determine how hard the players perceived the session (internal intensity and exercise load of the session) [34, 35]. These scores had been recorded during the two weeks prior to the investigation to ensure subjects familiarization with such tools. SSGs and RG were performed at the same time-of-day (from 16:00 to 18:00) to limit the effects of the circadian variations on the measured variables, particularly on HR measures [36].

### Statistical analysis

Data are presented as means and standard deviations (means ± SD). Before using parametric statistical test procedures, the normal distribution of data was verified. A two-way analysis of variance (ANOVA) ([periods] × [games]) with repeated measures was used to test for differences in performance measures (dependent variable) between the different periods within each SSGs (SSG2, SSG3) or the RG, independent variable. When the ANOVA indicated significant factor or interaction effects, a Bonferroni-corrected post hoc test was applied to investigate specific differences. The Shapiro-Wilk’s test was used to verify normal distribution. Effect sizes (Cohen’s d) for pairwise comparisons were calculated with the pooled standard deviations and considered as small (> 0.2–0.6), moderate (> 0.6–1.2), large (> 1.2–2.0), very large (> 2.0–4.0) and extremely large (> 4.0) [37]. All statistical analyses were performed using the software package STATISTICA (StatSoft^®^, Maisons-Alfort, France) and statistical significance was set, *a priori*, at P < 0.05.

## RESULTS

### HR responses

**FIG. 2:**
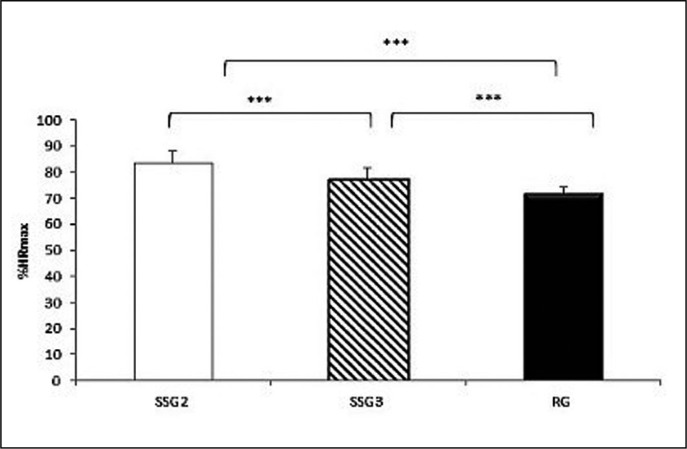
Percentage of maximum heart rate (%HR_max_) during SSG2, SSG3 and RG. *** significant difference at P < 0.001.

The one-way ANOVA showed a significant effect of game format (F_(2,22)_ = 37.64; P < 0.001; ƞ_p_^2^ = 0.77) on the %HR_max_. Post-hoc analysis showed that the %HR_max_ was significantly higher during SSG2 compared to the SSG3 (83.45 ± 4.06% vs. 77.12 ± 2.51%; P < 0.001; d = 1.41) and to the RG (83.45 ± 4.06% vs. 71.67 ± 4.55%; d = 2.41). Moreover, %HR_max_ was significantly higher during SSG3 compared to RG (77.12 ± 2.51% vs. 71.67 ± 4.55%; P < 0.001; d = 1.16) (Figure 2). The coefficient of variation for the %HR_max_ during different games formats is presented in table 1.

**TABLE 1:** Coefficient of variation for the physiological parameters during SSG formats

Parameters	SSG formats		
	2 vs. 2	3 vs. 3	6 vs. 6
**%HR_max_**	5.1%	3.4%	6.6%
**RPE**	8.6%	18.8%	17.1%
**[La^-^]**	58.0%	57.7%	53.8%

### RPE scores

**FIG. 3:**
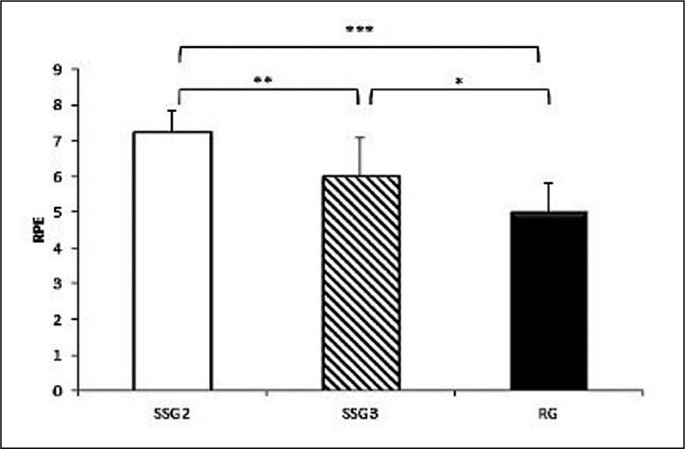
RPE values during SSG2, SSG3 and RG *** significant difference at P < 0.001, ** significant difference at P < 0.01 and * significant difference at P < 0.05.

The one-way ANOVA showed a significant effect of game format (F_(2,22)_ = 20.75; P < 0.001; ƞ_p_^2^ = 0.65) on the RPE. Post-hoc analysis results revealed that RPE scores were significantly higher during SSG2 compared to the SSG3 (7.25 ± 0.59 vs. 6 ± 1.08; P = 0.005; d = 0.92) and to the RG (7.25 ± 0.59 vs. 5 ± 0.81; P < 0.001; d = 1.98). Moreover, RPE was significantly higher during SSG3 compared to RG (6 ± 1.08 vs. 5 ± 0.81; P = 0.027; d = 0.89) (Figure 3). The coefficient of variation for RPE scores during different games formats is presented in table 1.

### Blood lactate concentration

**FIG. 4:**
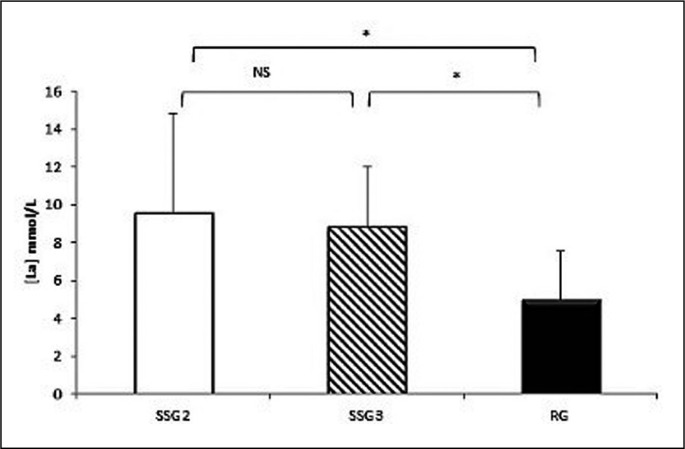
Lactate concentrations [La^−^] during SSG2, SSG3 and RG * significant difference at P < 0.05; NS: no significant difference.

The one-way ANOVA showed a significant effect of game format (F_(2,22)_ = 4.80; P = 0.019; ƞ_p_^2^ = 0.30) on [La^−^]. Post-hoc analysis results of the [La^−^] reported that there was no significant difference between SSG2 and SSG3 (9.54 ± 5.3 vs. 8.82 ± 3.18 mmol/l; P = 0.65). However, the [La^−^] was significantly higher in SSG2 (9.54 ± 5.3 vs. 4.99 ± 2.57 mmol/l; P = 0.026; d = 0.75) and SSG3 (8.82 ± 3.18 vs. 4.99 ± 2.57 mmol/l; P = 0.045; d = 1.07) compared to RG (Figure 4). The coefficient of variation for blood lactate concentration during different games formats is presented in table 1.

## DISCUSSION

The purpose of this study was to investigate the physiological (HR and [La^−^]) and perceptual (RPE) responses of SSGs and RG in youth volleyball players. Compared to RG, all physiological and perceptual responses (i.e., HR, [La^−^] and RPE) were significantly higher during SSG3 and SSG2. Moreover, HR and RPE were significantly higher in SSG2 compared to SSG3. In contrast, no significant differences were observed in [La^−^] between SSG2 and SSG3.Given the lack of studies on the subject of intensity in SSG in volleyball [38], the results of the present study will be compared with (i) those of team sports and (ii) the specificities and requirements of volleyball.

The results of this study demonstrate that SSGs provide greater physiological responses than RG. Indeed, %HR_max_ recorded during SSG2 and SSG3 was significantly higher than those of RG (83.45% vs. 77.12% vs. 71.67%, respectively). Further, these results are supported by those of Dellal et al. [15] and Jones and Drust [3] in soccer players, who showed that players presented higher %HR_max_ during SSGs vs. RG. The higher %HR_max_ recorded during SSGs could be explained by the greater technical, physical and tactical implication of all players both in offensive and defensive phases induced by SSGs. These results are likely due to the increase of shifting distance, the reduction of recovery period, and a higher incentive for players in SSGs vs. RG.

It is noteworthy that HR responses to exercise are generally not considered as the best indicator to examine the physiological requirements during SSGs. Indeed, the RPE has been used as an alternative measure of the soccer-training load [39], which could allow the analysis of the global internal load [3, 34]. The results of the present study showed that RPE values were higher in SSG2 and SSG3 than RG (7.25 and 6 vs. 5 A.U, respectively). Our results are in contrast with those of Dellal et al. [15] who found that RPE values were lower during SSGs. Indeed, RG appeared to be perceived easier than all SSGs situations. It would be expected from this data that the volleyball rule could induce a lower intensity within RG both concerning the physiological activities, which explain that players prefer this type of training.

Interestingly, in spite of the high-intensity of play observed during SSGs of the present study, [La^−^] was higher during the SSGs than during RG (9.54 vs. 8.82 vs. 4.99 mmol/l, respectively to SSG2, SSG3 and RG). Indeed, low values of [La^−^] during the RG in our experiment is the effect of natural game characteristics (serving and changing position). It results in recovery and rather low level of [La^−^] values. In general, in a volleyball game low [La^−^] (2.54 ± 1.21 mmol/l) during and after matches and increases of free fatty acids indicates that energy during the short exercise periods is mainly supplied by a breakdown of creatine phosphate, while aerobic pathways restore the energy sources during rest periods [40]. Due to the higher intensity performed during the SSGs, the anaerobic energy turnover would be expected to contribute more to the muscle metabolism in SSGs as compared to RG. In contrast to our findings, Dellal et al. [15] found, in soccer players, a higher [La^−^] within RG than SSGs. Dellal et al. [15] explained the lower [La^−^] recorded in soccer SSGs by the short duration of resulting in a higher reliance on ATP and CP breakdown rather than anaerobic glycolysis.

SSGs are often used as part of regular training programs in various forms, depending on the aim and the philosophy of the coach [41]. Moreover, it allows more time spent managing the ball under game-like conditions compared with generic training [25, 42]. Thus, most exercise sessions in team sports have SSG played with a reduced number of players on a smaller area than the regular official game pitch size [39]. Given that it has been shown that the physiological responses during SSGs are higher than within RG, it is feasible to suggest that SSGs may be used as a physical training modality for volleyball [24]. Indeed, all measured physiological responses in the present study provide informative detail, but it is important to study these physiological results within different SSG formats and pitch dimensions.

This study extends the findings of others demonstrating that a higher number of players can significantly alter the physiological responses during SSGs [39, 43, 44]. In the present study, SSG2 had the highest %HR_max_ (83.45%) compared with SSG3 (77.12%). In this context, Dellal et al. [45] and Rampinini et al. [39] investigated the effect of changing number of players in football on HR responses in different conditions. The authors observed a higher %HR_max_ and greater HR reserve with a reduced number of players. These findings are comparable to the results of Casamichana et al. [10] who revealed that a larger area per player determines a higher effective playing time, %HR_max_, time spent above 90% HR_max_, and RPE. The highest %HR_max_ reported in our study could be explained such that the recovery for the inactive player during SSG3 meant more tactical combinations could be performed. Accordingly, this may increase active recovery, and thus prepare the players for high intensities and to restart in the subsequent offensive or defensive process. Indeed, these results can be explained by the greater number of opponents in SSG3 that increases the uncertainty of the player regarding their actions.

In general, studies have shown that SSG formats with fewer players elicit greater RPE than the larger formats in soccer [34, 39, 46, 47, 48]. The latter findings are concordant with our results, where SSG2 induced significantly higher RPE responses compared to SSG3 (7.25 vs. 6 A.U respectively). These authors explained that the increase of pitch ratio per player induced higher RPE values, probably due to the increase of running distance, the greater activity, the reduction of recovery period within the SSG, and that players were systematically more stimulated. The decrease of RPE within SSG3 in our results can be explained by the collaboration between players allowing an inactive recovery. In addition, increasing the numbers of player can promote the teamwork and stimulate the partnership.

Unlike previous studies [1, 15, 49], we showed no significant differences in [La^−^] between SSG2 and SSG3 (9.54 vs. 8.82 mmol/l, respectively). This could be explained by the characteristics of the volleyball game; in both SSG2 and SSG3 format, players are quite free to move and change their position. Furthermore, since the specificity of volleyball requires much more placement than movement, the two forms of SSGs require the same time motion where the players must imperatively participate in each action, where, 0, 1 or 2 players are on block and 1, 2 or 3 in the back defense [25]. The specificity of this sport obliges the defender to cover a wider space (forward and backward movement) for the 1 vs. 1 game as it also forces the blocker to block 2 spikers across the net (lateral or cross movement). These findings are useful for coaching and developing the technical skills of novice players because it can promote a higher individual participation and stimulate the technical and physiological aspects.

Concerning the small number of players participating in this study (n = 12), most studies in SSG have used a small number of subjects in their research, and many of them have used the same number as of our sample size [22]. Moreover, in the present study, 12 players fulfilled the inclusion criteria for participating in the research; which permitted all players to participate in the RG. Although the present study provides a novel addition to the literature concerning physiological responses in volleyball using two forms (SSG and RG) in youth players and the intensity of SSG in volleyball, there are some limitations that should be considered. First, the results cannot be generalized to all volleyball training and can only be applied to youth players. Future studies should compare the effects of RG with those of SSGs for physical and tactical learning of adult players and the collective organization of teammates. Furthermore, the lack of the use of video and GPS in this study precludes more accurate data on the players’ motion. Accordingly, further studies with RG and SSG should consider using GPS and video analysis to ascertain more detailed insight into specific movement patterns. In terms of practical implications, coaches should consider using instruments that allow them to monitor intensity among players during training. In addition, coaches can consider these two forms of SSG (2 vs. 2 and 3 vs. 3) as a part of training when planning. Finally, given that the coach was involved during the sessions, there are issues of potential bias given the personal relationships of the coach to the players, this causing artificial changes in motivation, extraneous to the study parameters. However, given that coaches are almost always involved in player training, it was deemed acceptable for the purposes of this study.

## CONCLUSIONS

The main findings of the present study suggest that small-sided games elicit higher physiological responses than regular game. In this context, it appears that the higher physiological responses of youth volleyball players during SSGs are linked with the greater involvement of each player in the game. Within small-sided games, changing the number of players elicited significant changes in heart rate and rating of perceived exertion, while there was no significant difference in blood lactate. Moreover, the high intensity of small-sided games 2 compared with small-sided games 3 is likely attributable to the pitch area per player. Finally, the results of this study could help coaches/practitioners in planning seasonal programs and multifunctional aspects of specific training sessions in youth volleyball players and to improve their physiological and/or technical training.
